# *S. lugdunensis* Native-Joint Septic Arthritis: Case Report and Review of the Literature

**DOI:** 10.1155/2017/8903907

**Published:** 2017-12-28

**Authors:** C. Danielle Tan, Donna Moritz, Alfredo J. Mena Lora

**Affiliations:** ^1^University of Illinois College of Medicine at Chicago, Chicago, IL, USA; ^2^University of Illinois at Chicago, Division of Infectious Diseases, Department of Medicine, Chicago, IL, USA

## Abstract

*Staphylococcus lugdunensis* is a skin commensal classified as a coagulase-negative *Staphylococcus* (CoNS). Though CoNS is typically associated with less aggressive clinical disease than *Staphylococcus aureus*, there is growing awareness that *S. lugdunensis* may be as virulent as *S. aureus*. The association between *S. lugdunensis* and infective endocarditis is well known, but few reports of native-joint disease with this organism exist. We report a case a 28-year-old male with no prior medical problems presenting with native-joint septic arthritis. Cultures grew *S. lugdunensis*. To our knowledge, this is the fifth case reported in the literature.

## 1. Introduction

*S. lugdunensis* was first described in 1988 [1]. It remains a rare pathogen, but it has since been associated with a wide variety of clinical infectious syndromes, including cardiovascular, soft-tissue, bone, and prosthetic joint infections. *S. lugdunensis* has been associated with higher severity and mortality than other coagulase-negative *Staphylococcus* (CONS) species, presenting with features more similar to disease caused by *S. aureus*. A case series with native valve infective endocarditis (IE) caused by *S. lugdunensis* reported mortality as high as 42% and surgical needs for more than 51% of cases [2–5]. For prosthetic valve IE, mortality exceeded 78%. Soft-tissue infections and pyogenic disease involving *S. lugdunensis* have also been reported and may be more common than once thought in community-acquired infections [6]. *S. lugdunensis* and *S. aureus* can infect joints via direct inoculation or hematogenous spread. The association between *S. lugdunensis* and prosthetic joint disease is well established, with reports of infections ranging between 6 weeks and 4 years after implantation [4, 7, 8]. There is a paucity of reports on infections involving native joints.

## 2. Case Presentation

A 28-year-old male with no past medical history presented to the emergency room with two weeks of right knee pain. He denied any recent sexually transmitted diseases, illicit drug use, or prior episodes of knee pain. He reports no trauma to the knee. Over-the-counter pain medications did not relieve his pain, which progressed and eventually limited his ability to use his joint.

**Figure 1:**
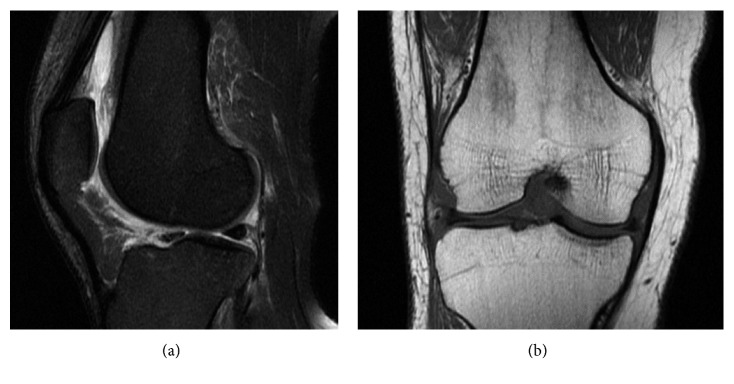
MRI of the knee in (a) T2 sagittal view and (b) T1 coronal view.

On examination, the patient was afebrile. His right knee was erythematous, warm, swollen, and with reduced range of motion. A moderate effusion was noticed. He was unable to bare weight on the affected leg. Laboratory analysis revealed leukocytosis, with white blood cell counts of 12,100 per microliter (mcL). Imaging revealed mild soft-tissue swelling and a moderate effusion. A diagnostic arthrocentesis was performed, and fluid analysis revealed 28,875 white blood cells per mcL, predominantly neutrophilic. Gram stain revealed Gram-positive cocci in clusters at 1 day of growth. At 48 hours, cultures grew *S. lugdunensis*. Magnetic resonance imaging (MRI) revealed diffuse soft-tissue edema, most prominent in the popliteal fossa around the knee and a large knee joint effusion with synovitis (Figure 1). Six synovial fluid samples grew *S. lugdunensis*. Susceptibilities were obtained via bioMerieux VITEK 2 antimicrobial susceptibility testing, showing resistance only to oxacillin.

The patient underwent washout by orthopedic surgery on day one and day three. Vancomycin was given for four days and subsequently changed to clindamycin. The patient completed seven days of parenteral antibiotics and transitioned to oral clindamycin for the remainder of a four-week course. He had full clinical resolution at his third week of therapy.

## 3. Discussion and Literature Review

Native-joint septic arthritis caused by *S. lugdunensis* is rare. Our review of the English literature found only four cases [7, 9–11]. One case did not have prior joint disease or chronic medical conditions, while all other cases had rheumatoid arthritis (RA) [7, 9–11]. Preexisting joint disease is a known risk factor for bacterial arthritis [12]. One prospective study of bacterial arthritis reported preexisting joint disease in 40% of cases, most commonly RA [13]. The most common pathogen associated with bacterial arthritis is *S aureus* [12]. However, the incidence of CoNS has risen in the past two decades and is more common with prosthetic joints. An observational study with 7275 total hip and knee arthroplasties over 38 years found 75 cases of prosthetic joint infections, with CoNS causing 18 infections, 3 of which were *S. lugdunensis* [14]. The incidence of CoNS increased from 10 to 21% during period [15].

*S. lugdunensis* is classified as a CoNS. It nevertheless retains partial coagulase activity and has many similarities with *S. aureus* [4, 8]. Agglutination tests are typically used to distinguish CoNS from *S. aureus*, but performance characteristics of these tests for *S. lugdunensis* are variable and can lead to false identification of *S. aureus* [4, 16]. Thus, the true burden of *S. lugdunensis* may be underappreciated. Samples may be reported as *S. aureus*, and both pathogens cause similar disease. Both organisms are skin commensals and are associated with soft-tissue infections, bacteremia, and infective endocarditis. Like *S aureus*, *S. lugdunensis* can cause severe disease. Infective endocarditis by *S. lugdunensis* can present with complications such as valvular perforation, myocardial abscesses, embolic stroke, and death [2, 4, 11]. Soft-tissue infections can be purulent and may progress to bacteremia and infective endocarditis [10, 11].

There are no randomized controlled data to guide the treatment of bacterial septic arthritis, and there is a paucity of data on *S. lugdunensis* infections. Three cases reported in the literature underwent joint irrigation, debridement, or other surgical interventions, and one did not specify (Table 1) [7, 9–11]. Antimicrobial courses included beta-lactams, glycopeptides, or lincomycins [7, 9–11]. Success with rifamycin combination therapy has also been reported [7, 9].

## 4. Conclusion

The present case contributes to a small but growing list of cases of native-joint septic arthritis by *S. lugdunensis*. Clinicians must be aware of the association between *S. lugdunensis* and severe clinical disease. Lincomycin-based therapy was a successful treatment option for our case. Further studies are needed to compare different therapeutic options.

## Figures and Tables

**Table 1:** Management of *S. lugdunensis*.

Case	Trauma or joint disease	Antimicrobial regimen	Duration in days	Surgical management
Begly et al. [9]	None	Vancomycin	28	Open medial parapatellar arthrotomy with irrigation and debridement on day 1
		Rifampin		
Grupper et al. [7]	None	Cefazolin	26	Daily joint aspiration from day 1–3
		Rifampicin		Arthroscopy with debridement on day 9 and 12
Kragsbjerg et al. [11]	Rheumatoid arthritis	Cloxacillin IV	42	Not specified
Rose et al. [10]	Rheumatoid arthritis	Flucloxacillin IV	112	Joint irrigation and washout
